# Unfinished agenda of the neonates in developing countries: magnitude of neonatal sepsis: systematic review and meta-analysis

**DOI:** 10.1016/j.heliyon.2019.e02519

**Published:** 2019-09-27

**Authors:** Desalegne Amare, Masresha Mela, Getenet Dessie

**Affiliations:** a Department of Pediatrics and Child Health, College of Medicine and Health Sciences, Bahir Dar University, 79, Bahir Dar, Ethiopia; b Felege Hiwot Referral Hospital, Bahir Dar, Ethiopia; c Department of Adult Health Nursing, College of Health Sciences, Bahir Dar University, Bahir Dar, Ethiopia

**Keywords:** Public health, Infectious disease, Pediatrics, Emergency medicine, Clinical research, Meta-analysis, Neonatal sepsis, Neonates, Systematic review, Developing countries, Sepsis

## Abstract

**Purpose:**

Neonatal sepsis is the major cause of mortality and morbidity globally, particularly in developing countries. Despite studies revealed the extent of neonatal sepsis in developing countries, the findings were inconclusive. Therefore, the main aim of this study was to determine the pooled prevalence of neonatal sepsis in developing countries.

**Methods:**

We used a systematic review and Meta-analysis study method. The reviewed studies were accessed through an electronic web-based search strategy from the electronic database (PUBMED), advanced google scholar, different journal sites. The data extraction was done by two researchers using a data extraction table and the disparity between data extractors was resolved by the third researcher. The analysis was done using STATA version 11. The I2 test was used to assess heterogeneity across studies. The Funnel plot, Begg's test, and Egger's test were used to check for publication bias. The random-effect model was used to determine the pooled effect size. All studies related to neonatal sepsis which fulfill the inclusion criteria were considered into this study. The quality of each study was checked using the Newcastle-Ottawa Scale and studies graded low score were excluded from the study.

**Results:**

At the end, 36 articles fit with our study objectives. Studies conducted in Ethiopia were significant the source of heterogeneity of the study with a coefficient = 90, P-value = 0.025. The overall pooled prevalence of the study was 29.92%. The limitations of this study would be the authors were only used articles reported in the English language, and publication bias.

**Conclusion:**

The pooled prevalence of neonatal sepsis was found to be high which accounted for a third of the neonates. Despite countries have established possible prevention and treatment mechanisms, neonatal sepsis is the major public health problem in lower and middle-income countries till now.

## 1 Introduction

In 2015, about 5.9 million under-5 death occurred [1], from 2 to 7 million have seen in the neonatal period [1, 2]. Of these, approximately 7000 newborns die every day, which accounted for 47% of all child deaths under the age of 5-years [2]. In general, about 99% of neonatal deaths occur in lower-income and middle-income countries. The remaining 1% of death was from resource-rich nations, and deaths in these countries did not attract sufficient attention from researchers, policy-makers and other key stakeholders. Indeed, many of them happen at home and were often unrecorded [3]. Conventionally, an estimated of 5.29–8.73 million disability-adjusted life years are lost annually in Sub-Sahara due to neonatal sepsis [4].

Sepsis is a major cause of mortality in the first month of life. Overall sepsis causes for 6.8% under-5 mortality from 2000–2015 [1]. The most common (81%) isolated bacteria were gram-negative [5]. A report showed that the incidence of neonatal sepsis was about 20.5%. Of these, *Staphylococcus aureus* accounted for the highest percentage (60%) followed by *Klebsiella pneumonia* (23%) [6]. On the other hand, gram-negative bacteria accounted for 78.9% of all isolates and were the only organisms encountered in early onset sepsis [7]. The incidence of neonatal sepsis was 10.3 per 100 admissions, which was based on blood culture-positive results for 196 out of 236 neonates [8]. Other report revealed that about 65% had presented with early onset neonatal sepsis and 22.4% of them had culture-proven sepsis [7]. Studies in Ethiopia showed that the prevalence of neonatal sepsis was 77.9% [9], 76.8% [10], 72.9% [11], 46.6% [12].

Although a systematic review and meta-analysis study was conducted in high and middle-income countries [13], there is no study conducted in lower-income countries. Therefore, this systematic review and meta-analysis study aimed to address this gap in the literature by determining the pooled prevalence of neonatal sepsis in developing countries.

## 2 Main text

### 2.1 Methods

#### 2.1.1 Eligibility criteria

##### 2.1.1.1 Inclusion criteria

• All studies related to neonates either admitted in the hospital or community-based studies within the neonatal period of life in African, Asian and Latin America was included in the study. These developing countries were Nigeria [7, 14, 15, 61], Nepal [16, 17, 18], Tanzania [19], Ethiopia [9, 10, 12, 20, 21], Egypt [22, 23], Bangladesh [24], Sudan [25, 26], Indonesia [27], Zambia [28], India [29, 30, 31, 32, 33] Sri Lanka [34], Uganda [35], Haiti [36], Kenya [37], Ghana [38], South Africa [8], Cameroon [39], Brazil [40], Mexico [41], Jamaica [42] and Nepal [17, 18].
• Publication year: All articles published from 2005 to 2018 are used for systematic review and meta-analysis.
• Design: All observational studies which assessed the neonatal sepsis in developing countries are included in the study.
• Publication status: All published literature were included in the study.
• Language: Only articles published literature reported in the English language were included.

##### 2.1.1.2 Exclusion criteria

Studies conducted by systematic review and meta-analysis and studies with methodologically unclear were excluded from this study. Also, articles published other than the English language were excluded from this study because this might cause poor understanding and translation bias.

##### 2.1.1.3 Study design, information sources and search strategy

This systematic review and meta-analysis method was used by considering the Preferred Reporting Items for Systematic Reviews and Meta-Analyses (PRISMA) guidelines [43]. We used google and google scholar search engines, electronic databases (Pub Med, CINAHL Plus, Hinari Access to Research for Health programme) and different journal sites (Africa Journals Online, Global Health journal, Academic Search, Directory of Open Access Journals). This study was conducted from October first, 2018 to 5 November 2018. The searching terms were pre-defined to allow a comprehensive search strategy which included in all fields within records and Medical Subject Headings (MeSH terms) were used to help expand the search in advanced PubMed search. We also used Boolean operator (within each axis we combined keywords with the “OR” operator and we then linked the search strategies for the two axes with the “AND” operator). The key terms used to search were "newborn OR infant OR infancy AND sepsis OR infection AND developing AND countries". Moreover, the cross-reference list was used to retrieve other related articles. Endnote reference manager software was utilized to collect and organize search outcomes and to remove duplication.

##### 2.1.1.4 Study selection

After a full abstract has been retrieved and reviewed, and the studies which meet the inclusion criteria would then be obtained and reviewed in full. The review process has been done by two reviewers, this helps to increase the reliability of the data selected. The disparity between these reviewers was resolved with a third reviewer (MM) whenever appropriate. Finally, we saved all reviewed studies that fulfill the inclusion criteria.

##### 2.1.1.5 Data extraction

The data extraction was done by two researchers using a data extraction table. This data extraction table includes the authors' name, publication year, study design, sample size, study participants, response rate, study methods, study prevalence, illegibility criteria, and the searching terms. The definition of the neonatal period was used to extract the data "regardless of gestational age, the neonatal period begins at birth and includes the first month of life [17, 18]”.

##### 2.1.1.6 Quality assessment and data collection

Studies were eligible for data extraction when they met the Newcastle-Ottawa Scale tool criteria in terms of enough sample size, clarity of research aims, appropriateness of design, recruitment, data collection, analysis and reporting of findings. When there was unclear abstract whether a citation is relevant or not, it was excluded for full-text retrieval. Then the full text of potentially eligible papers against the inclusion criteria was assessed. The relevance of the reviewed studies was checked based on their topic, objectives, and methodology. A preliminary assessment was made and some articles were excluded from the first step based on their topics and abstracts. After reviewing the full article, the score was given based on the Newcastle-Ottawa Scale [44].

##### 2.1.1.7 Publication bias and heterogeneity

Statistical heterogeneity across the studies was evaluated by using I^2^ statistic and the continuous and categorical Meta-regression analysis was performed to determine the potential sources of heterogeneity. The Egger's and Begg's tests were applied to evaluate the potential publication biases of the studies. The random effect model was used to examine statistically significant heterogeneity and the trim and fill analysis was done to assess the presence of publication bias.

##### 2.1.1.8 Outcome of interest

The outcome of interest was the pooled prevalence of sepsis among neonates in developing countries. This pooled prevalence was measured as the number of neonates with sepsis divided by the number of patients in a study multiplied by 100.

##### 2.1.1.9 Statistical analysis

We planned to analyze the pooled prevalence of neonatal sepsis using STATA software version 11. A forest plot was analyzed using Meta-regression analysis and significant heterogeneity was found within studies. A subgroup analysis was done to determine the heterogeneity within the regions by using the random-effects model. Begg's and Egger's tests were done to observe a publication bias. These Begg's and Egger's test with P < 0.05 were considered as significant publication bias. Finally, publication bias was assessed using the trim and filled analysis method.

### 2.2 Results

#### 2.2.1 Study selection

**Fig. 1:**
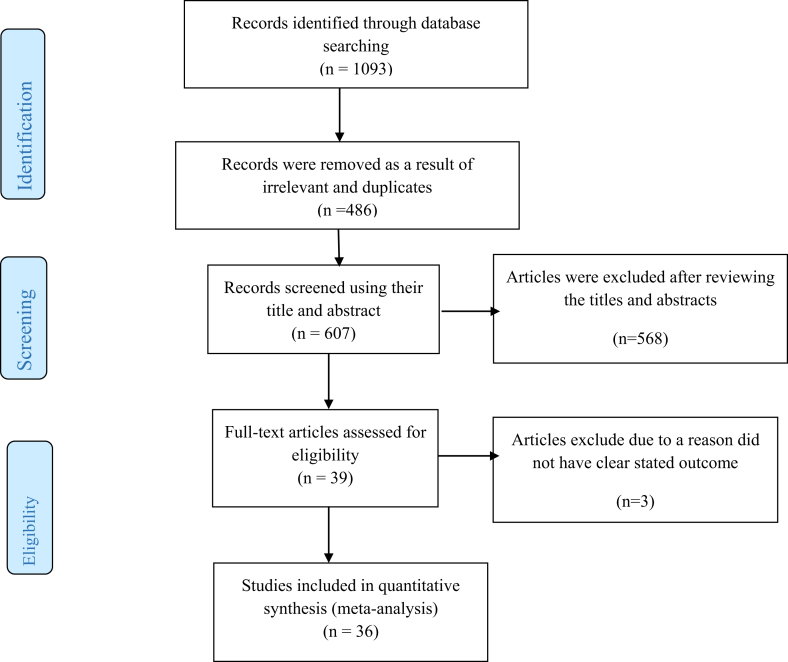
Flow diagram showing the procedure of screening studies for meta-analysis.

A total of 1093 Records were identified through the electronic database, search engines, and journal lists. Searching was conducted by the principal investigator and the co-author. From the total identified articles, 486 articles were excluded since they are duplicated. About 568 articles were removed by screening using their titles and abstracts. Three articles were excluded after using full-text review with the reason that the outcomes of the articles were not clear for researchers [10, 45, 46]. Finally, 36 articles fit with our study objectives (Fig. 1).

#### 2.2.2 Study characteristics

The maximum sample size obtained during searching was 34362 in India with retrospective study design [29]and the minimum sample size was 119 in Sudan along with cross-sectional study design [26]. The mean sample size of the study was 2226.8. Except one, all articles included in this study had 100% response rates. The majority (75.7%) of the studies were confirmed the neonatal sepsis through culture and the remaining articles diagnosis was settled using clinical signs and symptoms (Table 1).

**Table 1:** Characteristics of studies in Meta analysis of prevalence of neonatal sepsis in Africa, Asia, and Latin America.

Authors	Year	Study design	N	Response rate (%)	Diagnosis		Countries	PNS (95% CI)
					Culture/clinical	%		
Arowosegbe et al. [8]	2017	Cross-sectional	180	100	Culture positive	43.5	Nigeria	47.2 (39.65,54.75)
Thapa B et al. [17]	2014	Cross-sectional	300	100	Culture positive	17	Nepal	31.4 (24.64,38.16)
Jabiri A et al. [7]	2016	Cross-sectional	220	100	Clinical	---	Tanzania	77.9 (69.36,86.44)
Getabelew A et al. [11]	2017	Cross-sectional	244	100	Clinical	---	Ethiopia	8.6 (4.38,12.82)
Medhat H et al. [18]	2017	Retrospective cohort	1023	100	Clinical	8.6	Egypt	17.5 (11.89,23.11)
Raha BK et al. [19]	2014	Cross-sectional	720	100	Culture positive	8.9	Bangladesh	36 (28.98,43.02)
Kheir AEM et al. [12]	2014	Cross-sectional	354	100	Culture positive	61.3	Sudan	47.8 (30.67,44.92)
Hasibuan BS [22]	2018	Cross-sectional	626	100	Culture Positive	24.6	Indonesia	46.6 (39.07,54.13)
Kabwe M et al. [21]	2016	Cross-sectional	313	91.5	Culture positive	33	Zambia	43.5 (36.11,50.89)
Panigrahi P et al [22]	2017	Prospective cohort	842	100	Culture positive	100	India	34 (27.09,40.91)
Babiker W et al. [16]	2018	Cross-sectional	119	100	Culture positive	37.8	Sudan	76 (68.29,85.31)
G/eyesus T et al. [61]	2017	Cross-sectional	251	100	Culture positive	46.6	Ethiopia	67.9 (59.63,76.17)
Peterside O et al [23]	2015	Retrospective cohort	233	100	Culture positive	43.5	Nigeria	21.8 (15.76,27.84)
Sundarm V et al. [15]	2009	Retrospective cohort	34362	100	Culture positive	4.3	India	72.2 (63.8,80.6)
Agrawal A et al. [24]	2018	Cross-sectional	850	100	Culture positive	5.06	India	26.7 (20.26,33.14)
Perera KSY et al. [25]	2018	Case control	3482	100	Culture positive	2	Sri Lanka	45.9 (38.4,58.4)
Verma P et al. [26]	2015	Prospective cohort	3130	100	Culture positive	7.6	India	21.9 (15.85,27.95)
Shobowale OE et al. [27]	2016	Cross-sectional	250	100	Culture positive	34	Nigeria	10.3 (5.73,14.87)
Gebremedhin D ea al [12].	2016	Case control	234	100	Clinical	---	Ethiopia	34.7 (22.75,41.65)
Demisse AG et al. [28]	2017	Cross-sectional	769	100	Clinical	---	Ethiopia	38.1 (30.97,45.23)
John B et al. [29]	2015	Cross-sectional	174	100	Culture positive	21.8	Uganda	79.1 (70.53,87.67)
Boulos A et al. [30]	2017	Retrospective cohort	1292	100	Culture positive	74	Haiti	16 (10.57,21.43)
Minyahil AW et al. [31]	2014	Cross-sectional	306	100	Clinical	---	Ethiopia?	37.1 (30.04,44.20)
Kumar A et al. [32]	2010	Cross-sectional	310	100	Culture positive	26.7	Kenya	8.9 (4.62,13.18)
El-Din ERS [33]	2015	Retrospective cohort	778	100	Clinical	---	Egypt	24.6 (18.32,30.88)
Labi A-K et al. [34]	2016	Retrospective cohort	8025	100	Culture positive	21.9	Ghana	10 (5.49,14.51)
Shah AJ et al. [35]	2012	Prospective cohort	190	100	Culture Positive	31.6	India	4.3 (1.46,7.20)
Lebea MM et al. [9]	2017	Retrospective cohort	1903	100	Culture positive	10.3	South Africa	5 (1.88,8.24)
Chiabi A et al. [36]	2011	Prospective cohort	628	100	Culture positive	9.6	Cameroon	4.6 (1.61,7.59)
Ameyaw E et al. [37]	2017	Cross-sectional	1580	100	Clinical	---	Ghana	7.6 (3.62,11.58)
Emmanuel EN et al. [38]	2016	Cross-sectional	269	100	Clinical	---	Cameroon	31.6 (3.62,11.58)
Dal-Bó K et al. [39]	2012	Retrospective cohort	239	100	Culture positive	27.1	Brazil	12.6 (7.63,17.57)
Leal YA et al. [40]	2012	Retrospective cohort	11,790	100	Culture positive	16.9	Mexico	20.5 (14.58,26.42)
BELL Y et al. [41]	2005	Retrospective cohort	4702	100	Culture positive	2.9	Jamaica	54.8 (46.95,62.65)
Ansari S et al. [42]	2015	Cross-sectional	918	100	Culture positive	12.6	Nepal	45.8 (38.30,53.30)
Pokhrel B et al. [43]	2018	Retrospective cohort	336	100	Culture positive	20.5	Nepal	4.3 (1.44,7.16)
Olatunde OE et al. [44]	2016	Prospective cohort	450	100	Culture positive	16	Nigeria	2.9 (0.81,4.99)

#### 2.2.3 Prevalence of neonatal sepsis and heterogeneity

**Fig. 2:**
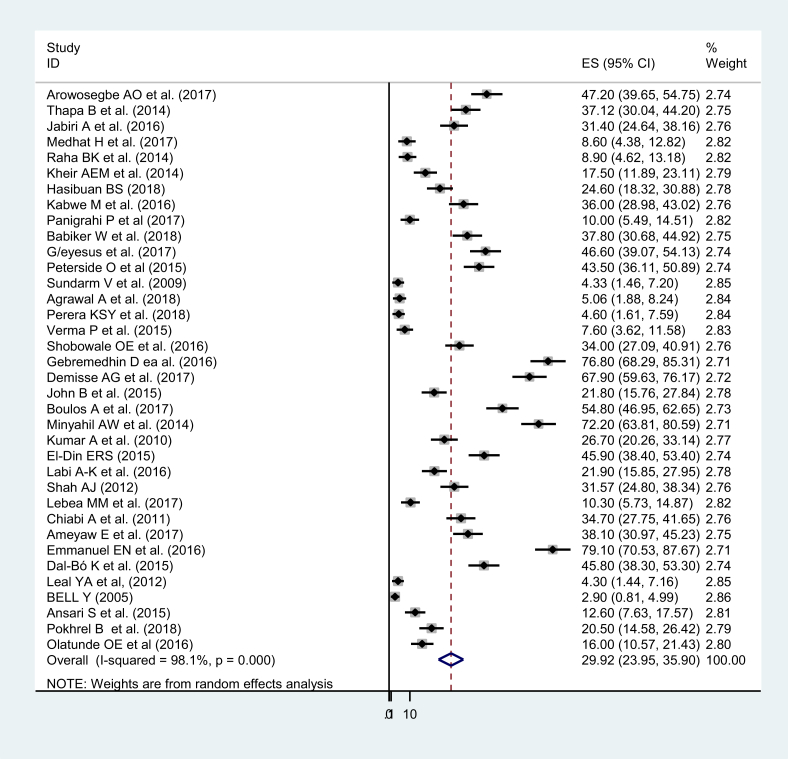
Forest plot, showing the results from a cumulative meta-analysis of 36 studies to determine the pooled prevalence of neonatal sepsis in developing countries.

**Fig. 3:**
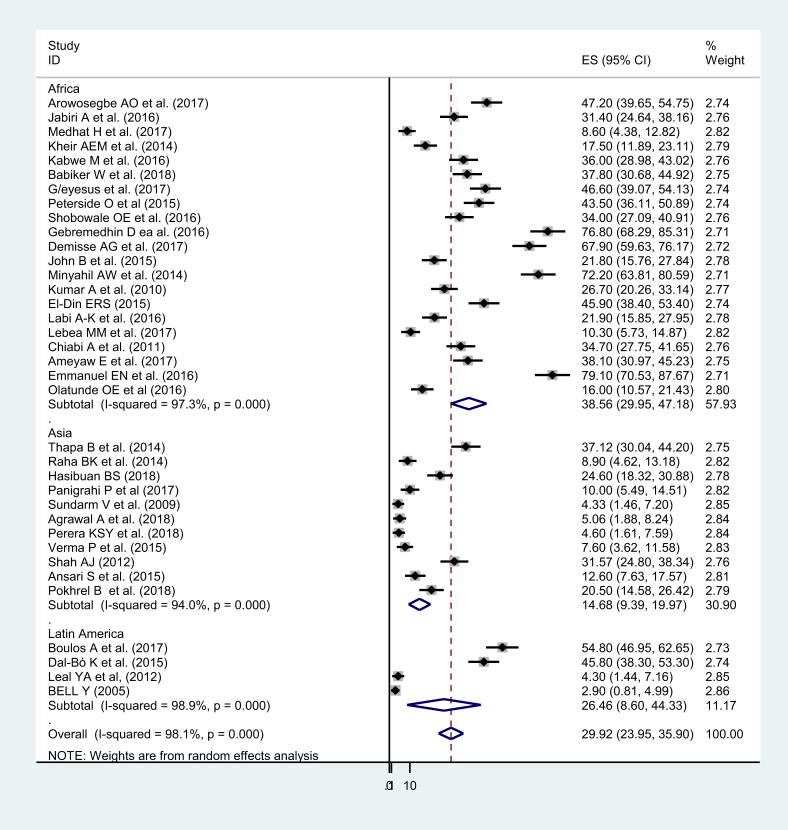
Subgroup analysis of the study by its regions.

The overall pooled prevalence was 29.92 with (95% CI 23.95, 35.90). The overall heterogeneity of this study was I^2^ = 98.1% (P-value = 000) (Fig. 2). The study was sub-group into three regions which are Africa, Asia, and Latin America. The high heterogeneity has shown within regions. The regional prevalence were 38.56, 14.68 and 26.48 in Africa, Asia, and Latin America, respectively. Heterogeneities in Africa Asia and Latin America were 97.3%, 94%, and 98.9 %, respectively (Fig. 3). Studies in Ethiopia have shown that there is a significant heterogeneity (coefficient = 90, P-value = 0.025) (Table 2).

**Table 2:** Meta-regression test on selected variables to identify source of heterogenity among studies.

Variables	Coefficient	P-value
Year of study	0.48	0.15
Sample size	0.25	0.17
**Institutions**		
District hospital	-49.1	0.33
General	-36.8	0.34
Referral	-54.6	0.28
Tertiary care hospital	-54.3	0.25
Tertiary	-51.5	0.30
Community	-48.1	0.38
**Study design**		
Case control	35.9	0.27
Cross-section	16.3	0.33
Retrospective	8.3	0.63
**Region**		
Bangladesh	23.9	0.52
Brazil	83.6	0.12
Cameroon	93.6	0.06
Egypt	68	0.20
Ethiopia	90	0.025
Ghana	66.7	0.16
Haiti	95.6	0.09
India	52.6	0.26
Indonesia	57.4	0.26
Jamaica	43.5	0.37
Mexico	42.1	0.41
Nepal	57.6	0.20
Nigeria	72.4	0.12
South Africa	50.9	0.30
Sudan	58.5	0.20
Tanzania	64.2	0.21
Uganda	63	0.35
Zambia	68.8	0.18
Kenya	59.5	0.24

#### 2.2.4 Risk of bias within studies

**Fig. 4:**
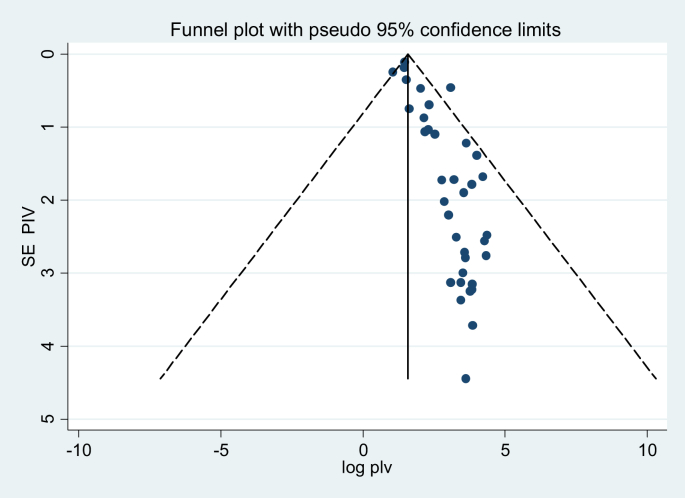
The funnel plots which are asymmetric and showed there are possible publication bias.

**Fig. 5:**
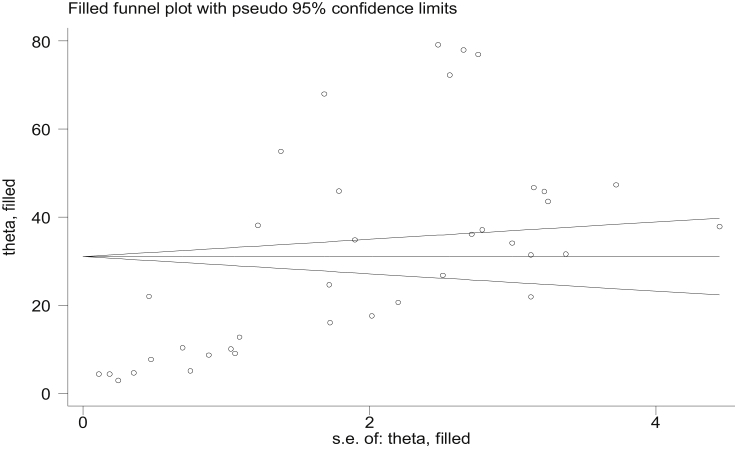
Filled funnel plot which has no shown publication bias.

The Begg's test has not shown a significant publication bias with (P-value>0.05). The Egger's test showed that there is a significant publication bias with P-value 0.001. The funnel plot test has shown that there are asymmetric plots. These plots indicated that there is a significant publication bias in which the majority of the plots were placed between 0 and +5 (Fig. 4). However, after a trim and filled analysis, publication bias has not been shown (Fig. 5).

### 2.3 Discussion

This systematic review and meta-analysis study was conducted to determine the pooled prevalence of neonatal sepsis in developing countries. We have found consistent evidence of higher levels of neonatal infection within 28 days with the pooled prevalence of 29.92% (95%CI 23.95, 35.90) which shows a major problem of the developing countries. This finding was appreciably higher than other studies conducted elsewhere 17% in China [47], 7.6% in India [48] and the prevalence of early-onset neonatal sepsis which was confirmed by the laboratory was 17.2% [49]. Even if the health care units advanced recently, sepsis remained the major causes of morbidity and mortality for neonates [50] and greater than 40% of under-five deaths occur in the neonatal period, resulting in 3.1 million newborn deaths each year globally [51]. The neonatal morbidity was predominantly higher in low and middle-income countries [52] particularly, in Africa and it is the third common cause of death [53, 54]. This difference could be due to a lack of well-established the health care system since the majority of studies have been taken in Sub Saharan Africa and another part of developing regions.

The prevalence of this study is consistent with the reports by Shah AJ et al [55] 31.57%, Jabiri A et al. [19], which accounted 31.57% and 31.4%, respectively. This high magnitude is the major public health issue in developing countries (Sub Saharan Africa), an estimated magnitude of range from 380 000–2 000 000 annual cases and 270 000 annual associated deaths [56]. Sepsis is one of the three most common causes of neonatal deaths globally [57]. Most infection in the neonatal period occurs in low and middle-income countries due to poor hygiene and suboptimal practices for infection control [58]. This can be the future agenda of the developing countries.

Majority of studies included in our study were confirmed through blood or cerebrospinal fluid culture [8, 14, 16, 18, 19, 24, 26, 27, 29, 33, 36, 38, 39, 42, 48, 55, 59, 60] and others were neonatal sepsis diagnosed using clinical signs and symptoms [9, 12, 20]. Consequently, studies with neonatal sepsis diagnosed using clinical signs and symptoms of infection may have a low magnitude of the association compared to studies confirmed with culture or laboratory test. This is because clinical diagnosis is less specific to settle the definite diagnosis of neonatal sepsis. In the other way diagnosis of neonatal sepsis in the early onset period, the result may undermine the true risk of the infection since the sensitivity of the result depends on the specimen collection process [61].

This systematic and Meta-analysis revealed that there was a significant heterogeneity throughout the studies within intern-regional and intra region. The source of heterogeneity could be studies conducted in Ethiopia because the Meta-regression of studies in Ethiopia has a significant P- value less than 0.05. Other reports have also supported that they have heterogeneity between studies [49] and consider this heterogeneity existed between studies given the various definitions of laboratory-confirmed and clinical signs of infection, as well as for colonization and risk factors [62, 63, 64].

Bacterial pathogens such as *Klebsiella, CoNS*, and *S. aureus* were the common cause of neonatal infections in the developing countries [7, 8, 14, 16, 17, 18, 19, 24, 26, 27, 29, 30, 33, 36, 38, 39, 42, 48, 55, 59, 60]. Congruently, it is supported by reports of the newborn problem in lower and middle-income countries [65, 66] and the evidence from other systematic review showed that *Klebsiella* species, *E. coli,* and *S. aureus* were the major cause of neonatal infection during the neonatal period [67]. This may be due to the susceptibility of neonatal population, lack of consensus in the definitions and pathogen variability between different regions which affect the development of clinical trials and practice guidelines [65].

The strength of this study was included different regions of lower and middle-income countries and we used extensive searching strategies to minimize the chance of missing the relevant articles and literature. For this systematic review and meta-analysis, using only articles reported in the English language was our limitation. Also, publication bias is the limitations of this study.

## 3 Conclusion

We concluded that the prevalence of neonatal sepsis was significantly higher among developing countries. The developing countries accounted for a third of the neonatal sepsis. Majority of neonatal sepsis were in Africa region. Bacteria is the leading cause of neonatal sepsis. Heterogeneity among studies was reported and can be existed between studies given the various definitions of laboratory-confirmed and clinical signs of infection, as well as for colonization and risk factors. Despite various countries have established a possible prevention and treatment mechanisms, neonatal sepsis is the major problem of lower and middle-income countries.

## Declarations

### Author contribution statement

All authors listed have significantly contributed to the development and the writing of this article.

### Funding statement

To conduct this research we have not received any specific grant from funding agencies in the public, commercial, or not-for-profit sectors.

### Competing interest statement

The authors declare no conflict of interest.

### Additional information

No additional information is available for this paper.
